# Association of ambient air pollution and pesticide mixtures on respiratory inflammatory markers in agricultural communities

**DOI:** 10.1088/2752-5309/ad52ba

**Published:** 2024-06-25

**Authors:** Matthew L Hughes, Grace Kuiper, Lauren Hoskovec, Sherry WeMott, Bonnie N Young, Wande Benka-Coker, Casey Quinn, Grant Erlandson, Nayamin Martinez, Jesus Mendoza, Greg Dooley, Sheryl Magzamen

**Affiliations:** 1 Department of Environmental and Radiological Health Sciences, Colorado State University, Fort Collins, CO, United States of America; 2 Department of Statistics, Colorado State University, Fort Collins, CO, United States of America; 3 Department of Environmental Studies, Dickinson College, Carlisle, PA, United States of America; 4 Department of Mechanical Engineering, Colorado State University, Fort Collins, CO, United States of America; 5 Central California Environmental Justice Network, Fresno, CA, United States of America

**Keywords:** ambient air pollution, organophosphate pesticides, respiratory health, leukotrienes, environmental mixtures, bayesian kernel machine regression, central california

## Abstract

Air pollution exposure is associated with adverse respiratory health outcomes. Evidence from occupational and community-based studies also suggests agricultural pesticides have negative health impacts on respiratory health. Although populations are exposed to multiple inhalation hazards simultaneously, multidomain mixtures (e.g. environmental and chemical pollutants of different classes) are rarely studied. We investigated the association of ambient air pollution-pesticide exposure mixtures with urinary leukotriene E4 (LTE4), a respiratory inflammation biomarker, for 75 participants in four Central California communities over two seasons. Exposures included three criteria air pollutants estimated via the Community Multiscale Air Quality model (fine particulate matter, ozone, and nitrogen dioxide) and urinary metabolites of organophosphate (OP) pesticides (total dialkyl phosphates (DAPs), total diethyl phosphates (DE), and total dimethyl phosphates (DM)). We implemented multiple linear regression models to examine associations in single pollutant models adjusted for age, sex, asthma status, occupational status, household member occupational status, temperature, and relative humidity, and evaluated whether associations changed seasonally. We then implemented Bayesian kernel machine regression (BKMR) to analyse these criteria air pollutants, DE, and DM as a mixture. Our multiple linear regression models indicated an interquartile range (IQR) increase in total DAPs was associated with an increase in urinary LTE4 in winter (*β*: 0.04, 95% CI: [0.01, 0.07]). Similarly, an IQR increase in total DM was associated with an increase in urinary LTE4 in winter (*β*:0.03, 95% CI: [0.004, 0.06]). Confidence intervals for all criteria air pollutant effect estimates included the null value. BKMR analysis revealed potential non-linear interactions between exposures in our air pollution-pesticide mixture, but all confidence intervals contained the null value. Our analysis demonstrated a positive association between OP pesticide metabolites and urinary LTE4 in a low asthma prevalence population and adds to the limited research on the joint effects of ambient air pollution and pesticides mixtures on respiratory health.

## 1. Introduction

California’s Central Valley consistently fails to meet National Ambient Air Quality Standards (NAAQS) for multiple criteria pollutants [1]. Most of the counties in the Central Valley have been in non-attainment for NAAQS for many years [1]. In particular, Fresno and Tulare Counties have both been in nonattainment for the 8-hour ozone (O_3_) (2015) standard from 2018 to 2023, and both counties have also been in nonattainment for the particulate matter (PM_2.5_) (2012) standard from 2015 to 2023 [1]. The Central Valley is a critically important agricultural region, producing forty percent of the United States’ fruit and nuts [2]. Due to extensive crop production in the region, residents of Fresno and Tulare Counties have a comparatively high exposure to agricultural pesticides. In 2017 and 2018, Fresno and Tulare Counties ranked 1st and 3rd, respectively, in California for the largest quantity of pesticides used (in terms of total pounds of active ingredients reported) [3]. California uses more pesticides than any other state in the US, applying 95 million kg of active ingredients throughout the state in 2018 [3].

A large body of evidence indicates that exposure to ambient air pollution is associated with adverse respiratory health outcomes such as decreased lung function and asthma [4, 5], as well as overall increased risk of mortality from lung cancer and cardiopulmonary disease [6]. However, while agricultural pesticides are also considered an inhalation hazard, there is considerably less information on the impact of pesticide exposure on respiratory health outcomes compared to air pollution exposure. A number of occupational health studies have found that acute pesticide exposure is associated with adverse symptoms of respiratory disease [7–10]. In addition, the long-term U.S.-based Agricultural Health Study has consistently demonstrated an association between self-reported pesticide exposure, and wheeze and bronchitis among farmers and their spouses [11, 12]. Organophosphate (OP) pesticides in particular have been shown to adversely affect respiratory health. A study in an agricultural community in California showed an association between OP pesticide exposure and decreased lung function in early childhood, specifically decreased forced expiratory volume (FEV_1_) and forced vital capacity (FVC) [13]. Similarly, in a nationally representative sample of Canadian adults, urinary OP metabolites were associated with decreased lung function (FEV_1_, FVC, FEV_1_/FVC ratio, and forced mid-expiratory flow (FEF_25%–75%_)) [14]. A study on rural women workers in South Africa also suggested an association between exposure to OP pesticides and an increase in asthma-related cytokines [15]. Further, there is documented evidence of voluntary self-poisoning with OP pesticides (via ingestion, inhalation, and dermal exposure routes) in some communities in Iran, India and Sri Lanka as a method of suicide [16–18].

Historically, the effects of air pollutants and pesticides on respiratory health have been studied as distinct domains of exposure. However, at the community level, these exposures are often experienced as mixtures. Pollution mixtures are influenced by pollution source, topography, meteorology, solar radiation, and other environmental factors, leading to exposure patterns that vary spatially and temporally [19, 20]. Implementing statistical models to evaluate the health effects of environmental mixtures can elucidate both the individual effects of pollutants on the health outcome as well as the interactions that exist between chemicals in the mixture. These efforts will improve our understanding of the cumulative impacts that environmental and chemical pollutant (ECP) mixtures have on health endpoints. The Central Valley of California provides an ideal study area to explore environmental mixtures as residents are exposed to high levels of both criteria air pollutants and pesticides.

The primary aim of this community-based study was to evaluate the association between ambient air pollution and agricultural pesticide mixtures and respiratory health outcomes, as measured by leukotriene E4 (LTE4) concentrations from urine samples. Specifically, including OP pesticide exposure in the ECP mixture may help to improve understanding of OP pesticide association with respiratory health. Secondarily, we sought to investigate how this environmental exposure mixture-respiratory health relationship changed seasonally, using measurements from two unique periods in the year when the pollution mixtures have different compositions and properties.

## 2. Methods

### 2.1. Study location and participants

The Study of Environmental Mixtures in Periurban Respiratory Outcomes (SEMIPRO) seeks to understand the relationship between ambient ECP exposures and respiratory health endpoints among residents of the Central Valley of California.

We partnered with the Central California Environmental Justice Network (CCEJN) to identify four study communities based on previous engagement projects and nearby active fields. Using Google Earth, CCEJN staff identified eligible residences that were within approximately 61 meters of cropland or orchards (i.e. agricultural fields). The four study communities were above the 92nd percentile for quantity of pesticides applied in 2018, according to data from the California Pesticide Use Report [21]. Outreach staff from CCEJN then conducted a door knocking campaign of eligible households to communicate face-to-face and enroll participants in the study. Participants who spoke English or Spanish, were able to provide a urine sample, and lived at their current address for at least one year were eligible to participate. Prior to participation in the study, we obtained consent of participants 18 years and older and assent for those younger than 18 years old, in addition to consent of their parent or guardian. We conducted a repeated measures panel study; study visits were conducted in 2019 during 1st–18th January, considered to be the low pesticide application season, and again during 1st–13th June, considered to be the high pesticide application season. The Colorado State University Institutional Review Board approved all study procedures.

### 2.2. Survey and sampling methods

The SEMIPRO team conducted all study visits in person and administered a survey to participants in either English or Spanish to gather information on characteristics of the home (e.g. ventilation, number of windows, number of doors), socioeconomic status (e.g. level of educational attainment), health status, and workplace exposures [21]. The first participant interviewed in each home answered questions about household features; all subsequent participants only answered questions related to individual factors. We entered the survey responses into REDCap electronic data capture tools hosted by the Colorado Clinical & Translational Sciences Institute.

Consenting and assenting participants in the household also provided urine samples. Participants followed instructions to wash their hands, use a sanitary wipe to clean the perineal area, fill the sample collection cup halfway, and return the sample to study staff in biohazard bags. After collection, we stored urine samples at −80 °C at California State University, Fresno. At the end of each campaign, we shipped the samples overnight on dry ice to our lab in the Environmental Health building at Colorado State University (CSU). The samples remained frozen at −80 °C until they were thawed at 4 °C for 24–48 hours and processed for analysis.

#### 2.2.1. Pesticide exposure

Dialkyl phosphate metabolites (DAPs) concentrations in the urine samples (ng ml^−1^) were a primary exposure of interest for this analysis. DAPs are biomarkers of OP pesticide exposure typically formed during hydrolytic metabolism of OPs and are excreted in the urine [22]. There are six DAPs that are categorized into two classes: the dimethyl alkylphospate (DM) group includes dimethylphosphate, dimethlylthiophosphate, and dimethyldithiophosphate while the diethyl alkylphosphate (DE) group includes diethylphosphate, diethlythiophosphate, and diethyldithiophosphate [22]. Each of these six DAPs can result from the metabolism of multiple OP pesticides that differ in their toxicity [23]. Therefore, the detection of DAPs in the urine cannot directly indicate the parent OP compound [22]. Here, we investigated total DAPs, total DM, and total DE concentrations as continuous exposure variables in statistical models.

The CSU Analytical Toxicology lab followed published methods and protocols for the DAP analysis [24, 25]. This method was validated following The Scientific Working Group for Forensic Toxicology guidelines and included bias and precision, calibration model, carryover, interferences, limit of detection, limit of quantitation, and stability. Matrix matched calibrators and controls were prepared by spiking DAPs into previously tested blank human urine. Briefly, we aliquoted urine samples (3 mls) into 10 ml glass test tubes with 20 *µ*l of an internal standard solution (10 *µ*g ml^−1^ each of DMP-D6, DMDTP-^13^C2, DETP-^13^C4, DEP-^13^C4, DEDTP-D10, and DMTP-D6) and frozen at −80 °C overnight. We lyophilized frozen urine samples overnight using a Labconco FreeZone 4.5 at −50 °C. We extracted DAPs from lyophilized urine samples by sonication in 5 ml acetonitrile for 10 minutes followed by 2 hours on a rotary mixer. We centrifuged samples at 3200 rpm for 5 minutes, transferred supernatants to clean 10 ml test tubes, and dried them under nitrogen at 40 °C until ∼500 *µ*l were left. We mixed the samples with 1.5 ml of 0.1% formic acid in water and transferred them into amber glass autosampler vials for LC-MS/MS analysis.

We analysed prepared calibrators, controls, and samples with an Agilent 1290 UHPLC coupled to an Agilent 6460 triple quadruple mass spectrometer equipped with an Agilent Jet Stream electrospray ionization source (Agilent, Santa Clara, CA). We first chromatographically separated DAPs on a Phenomenex Gemini NX-C18 column (2.0 × 150 mm, 3 *μ*m) held at 40 °C. We injected a sample volume of 5 *μ*l and a mixture of 99% water with 0.1% formic acid (A) and 1% acetonitrile with 0.1% formic acid (B) at a flow rate of 0.4 ml min^−1^. The gradient elution used was 1% B for 2 minutes then to 65% B at 8 minutes. The ionization source conditions used were as follows: nebulizer 40 psi; gas flow of 10 l min^−1^ at 300 °C; sheath gas flow of 11 l min^−1^ at 390 °C. We set the electrospray ionization negative to positive for all analytes. We monitored two ion transitions (m/z) for each analyte and internal standards. These ion transitions and corresponding fragmentor and collision energy voltages are displayed in supplementary table 1. We confirmed compound identifications by retention time and the product ion ratios (±20%). The data collection and processing were performed by using Agilent MassHunter Quantitative software (v.B.08.01). Isotope dilution and linear regression were used to quantitate urinary concentrations of DAPs using 6-point calibration curves from 0.5 ng ml^−1^ to 200 ng ml^−1^ for DMP, DMTP, DMDTP and DETP. For DEP and DEDTP, a 6-point calibration curve from 1 ng ml^−1^ to 200 ng ml^−1^ was used. The limits of detection for each DAP compound were as follows: 0.1 ng ml^−1^ for DMP, 0.1 ng ml^−1^ for DMTP, 0.1 ng ml^−1^ for DMDTP, 0.4 ng ml^−1^ for DEP, 0.1 ng ml^−1^ for DETP, and 0.4 ng ml^−1^ for DETDP. Blank urine samples were spiked with all DAPs at 25 ng ml^−1^ and extracted with the same methodology as field samples for quality control purposes. These samples were analysed following every 20 field samples with expected accuracy to be +/−15%. The coefficient of variation was <10 for all concentrations.

#### 2.2.2. Air pollution exposures

We extracted air pollutant concentrations from simulations performed with the Community Multiscale Air Quality Model (CMAQ) version 5.3.1 [26]. CMAQ, an air quality modeling system developed and managed by the Environmental Protection Agency, is a three-dimensional, regional chemical transport model that simulates the emissions, transport, chemistry, and deposition of air pollutants and predicts the spatiotemporal distribution of trace gases and particles in the free troposphere [27]. Full details about the CMAQ model, the inputs used to perform the simulations, and evaluation of model output can be found in Appel *et al* [26]. Briefly, using meteorological inputs from the Weather Research and Forecasting model and emissions inputs from the National Emissions Inventory, CMAQ simulates air quality over the contiguous United States at a grid resolution of 12 × 12 km^2^. Model predictions of O_3_, PM_2.5_, and NO_2_ from this version of CMAQ compared favourably with ground observations from EPA’s routine air quality monitoring network [28], with a slight negative bias in O_3_ and PM_2.5_ for the Southwest United States, including California.

Each of the four communities included in this study are contained within a unique and separate CMAQ grid cell. Therefore, ambient air pollution exposures were assigned at the community level. To estimate community-level ambient air pollution exposures for O_3_, NO_2_, and PM_2.5_, we first summarized January and June 2019 CMAQ hourly data for the relevant 12 × 12 km^2^ grid cell to daily concentrations according to NAAQS standards. For O_3_, we assigned each day the maximum daily 8 hour concentration (MDA8). For PM_2.5_, the daily concentration was the average of all hourly concentrations over the 24 hour period. Finally, daily NO_2_ concentrations were assigned as the maximum hourly value of each day. Then, for each pollutant, we calculated a monthly exposure for January and June as the average of the daily concentrations. We assigned these community-level average monthly pollutant estimates to the entire community for the two different months of the study.

#### 2.2.3. Outcome variable

Leukotriene E4 (LTE4) urinary concentration (pg/mg of creatinine) was the outcome variable of interest in this study. LTE4 is a metabolite of cysteinyl leukotrienes (cys-LT), produced by cells associated with asthma- and/or allergen-induced respiratory inflammation (i.e. eosinophils, mast cells, and basophils) [29, 30]. LTE4 is an established biomarker for detecting respiratory inflammation in people with asthma [30–32]. However, cys-LT have also been found in the exhaled breathe condensate of healthy individuals [33].

Isolating LTE4 from urine is a sensitive and non-invasive method for assaying production of total body cys-LT and has been described [34] in multiple studies [35–37].

We measured LTE4 concentrations in urine samples collected for each field campaign using ELISA kits from Cayman Chemical (Ann Arbor, MI) Kit #501060, and used the Cayman Creatinine Colorimetric Kit to normalize with urinary creatinine concentrations.

#### 2.2.4. Covariates

To identify factors that influenced the environmental exposures and health outcome of our study, we employed a data-driven best subsets regression approach to model building; this process has been described in a previous study [21]. Next, we conducted a Wilcoxon signed-rank test of the potential confounder and the outcome to evaluate associations (see supplementary table 4). Ultimately, we considered seven covariates: age (continuous), biological sex (categorical), employment in agriculture (categorical), any household member employed in agriculture (categorical), self-report of asthma diagnosis (categorical), monthly average daily maximum ambient air temperature (continuous), and monthly average relative humidity (continuous). The five individual-level covariates were derived from the SEMIPRO surveys that were administered to each participant. The air temperature and relative humidity covariates were obtained from the Automated Surface Observing Systems (ASOS) provided by the Iowa Environment Mesonet website [38], using the *riem* package in R. First, we identified the closest ASOS stations to each of the four study communities. If temperature and relative humidity data during the study period were missing for the closest monitor to the community, we identified the next closest station for which air temperature and relative humidity data for the study period were available. We then calculated the maximum daily air temperature and the average hourly relative humidity for each day, and assigned an average of the daily measurements to each community for January and June. Not all meteorological data were available for the entire months of January and June, so we utilized data from 1–18 January 2019, and 1–13 June 2019, that coincided with participant data collection.

### 2.3. Statistical analysis

#### 2.3.1. Descriptive analysis

We plotted outcome data and continuous exposure data to visualize their distribution, and to assess descriptively as frequencies and mean values. Based on non-Gaussian distributions of LTE4, we performed Wilcoxon (paired) signed-rank tests on each of the exposure variables to assess potential differences in outcome levels between January and June.

#### 2.3.2. Multiple linear regression analysis

We analyzed O_3_, NO_2_, PM_2.5_, total DAPs, total DM, and total DE individually as the main exposure variable in multiple linear regression models, with log-transformed LTE4 as the response variable. We fit three models for each pollutant. The first model only included the main exposure variable and the response variable. The second model included the five individual covariates: age, sex, asthma diagnosis, current employment in agriculture, and current employment in agriculture of any household member. The third model also included temperature and relative humidity as community-level covariates. Effect estimates were scaled to represent the change in log-transformed LTE4 concentration per an increase in exposure variable (air pollutant or pesticide metabolite concentration) equal to its interquartile range (IQR). We reported results as a percent change in outcome [100*(exponentiated regression coefficient −1)] per unit change in the independent/exposure variable.

#### 2.3.3. Bayesian kernel machine regression (BKMR)

We fit BKMR to estimate the association between exposure to the air pollution and pesticide mixture and the respiratory health outcome. BKMR is an advantageous method for analyzing mixtures because it estimates an exposure-response association by utilizing a flexible surface that allows for non-linear and non-additive interactions between mixture components [39]. The model also performs a hierarchical variable selection using prior knowledge of the exposure mixture’s structure [39, 40].

BKMR allows us to estimate the effects of individual pollutants in the mixture as well as interactions between those pollutants in the mixture, which may or may not be linear. Thus, both individual and joint effects of pollutants in the mixture can be assessed. Through variable selection BKMR estimates the relative contribution of each pollutant mixture component to the health outcome. In hierarchical variable selection the exposures can be partitioned into groups (e.g. pesticides and air pollutants).

Taking into account the possible correlations between exposure variables and the overall structure of a mixture, hierarchical variable selection estimates posterior inclusion probabilities (PIPs), which give the probability that exposure variables (the air pollutant and pesticide concentrations) are associated with the outcome (LTE4 concentrations) [39, 40]. This is done first by estimating the probability that a group (or domain) of pollutants should be included in the model (group PIPs), and then, conditional on group inclusion, assessing how each component of that group/domain drives the association with the outcome (conditional PIPs) [40].

The BKMR model is represented by the function:

\begin{equation*}{{y_{i} = h\left(x_{i}1, \ldots ,x_{i}M\right) + z_{i}^T\beta + \varepsilon_i}}\end{equation*}

where *y_i_* is the health outcome (LTE4 concentration), *x_i_* represents components of the exposure mixture (air pollutants and/or pesticides), *M* is the number of components in the exposure mixture, *h()* is a smooth exposure-response function allowing for non-linearity and/or interaction between mixture components, *z_i_* are model covariates (the same covariates used in the multiple linear regression models), and *ϵ_i_* are normally distributed residuals [40].

We fit BKMR separately for the January data and for the June data. We performed the BKMR analysis using the *bkmr* package in R version 4.2.2 [39]. Specifically, we used the kmbayes() function, which implements a Markov chain Monte Carlo algorithm to obtain samples from the posterior distribution. We inspected trace plots to ensure parameter convergence.

We fit the model with hierarchical variable selection and scaled all the exposures to have mean 0 and variance 1. We calculated posterior means, analogous to the beta coefficients in linear regression analysis, for O_3_, NO_2_, PM_2.5_, and DE and DM. We extracted PIPs from the fitted BKMR model that provided a measure of variable importance for each exposure [41].

To visualize the smooth exposure-response function h() (equation (1)), which is a high dimensional surface, we examined univariate and bivariate cross sections of the surface for the relationship between one or two exposures and the outcome, while holding the other exposures at a fixed percentile [42]. First, we examined the univariate relationship between each exposure and the outcome while all other exposures were held at their median. We also examined the bivariate exposure-response relationship for two exposures, for which all the other exposures were held at their 25th, 50th, and 75th percentiles. All analyses were performed in R version 4.2.2 [43].

## 3. Results

### 3.1. Descriptive statistics

We contacted a total of 200 households via a door-knocking campaign and ultimately recruited 80 participants across 34 households for the study. Three participants were lost to follow up and did not participate in the June campaign. Six participants in January, and five participants in June did not have urine samples with detectable LTE4. In total, LTE4 was extracted from 74 urine samples that were collected in January, and 75 urine samples that were collected in June. Participants who were lost to follow up or who did not yield a urine sample with detectable LTE4 were excluded from analysis. Study population characteristics are presented in table 1.

**Table 1.** Characteristics and demographics of study population at baseline.

Covariate	Community 1 (*n* = 32)	Community 2 (*n* = 5)	Community 3 (*n* = 23)	Community 4 (*n* = 15)	Total (*n* = 75)^a^
Male	12 (37.5%)	3 (60.0%)	10 (43.5%)	7 (46.7%)	32 (42.7%)
Age (years)					
Mean (SD)	40.4 (22.7)	53.2 (19.7)	41.1 (19.4)	42.0 (19.9)	41.7 (20.8)
Median [Min, Max]	35.5 [7.1, 87.8]	59.3 [18.9, 68.2]	46.6 [11.7, 81.3]	46.0 [13.7, 71.7]	46.0 [7.1, 87.8]
Self-reported asthma diagnosis	5 (15.6%)	0 (0%)	2 (8.7%)	4 (26.7%)	11 (14.7%)
Participant works in agriculture	8 (25.0%)	1 (20.0%)	9 (39.1%)	5 (33.3%)	23 (30.7%)
Household member works in agriculture	30 (93.8%)	2 (40.0%)	15 (65.2%)	14 (93.3%)	61 (81.3%)

^a^ Although 80 participants were recruited, 6 participants were excluded from January analysis, and 5 participants were excluded from June analysis, due to loss of follow up or lack of a urine sample with detectable LTE4. These numbers reflect the number of samples that were used in analysis for June (*n* = 75).

The majority of study participants were female (62.5%) and the mean age was 41.7 years old (standard deviation, SD: 20.8). Prevalence of asthma among the total study population was 14.6% (*n* = 11), nearly double the overall national asthma prevalence (7.6%) [44]. Stratifying by age, asthma prevalence was 12.3% among adults and 30.0% among children younger than 18 years of age. Over two-thirds of all study participants were working age (*n* = 52), defined as participants between the age of 18–64, and approximately 37% (*n* = 19) of the working age participants were employed in agriculture. Furthermore, 81% of all participants lived in a household in which at least one person worked in agriculture (*n* = 61).

### 3.2. Exposure and outcome summary statistics

**Figure 1.**
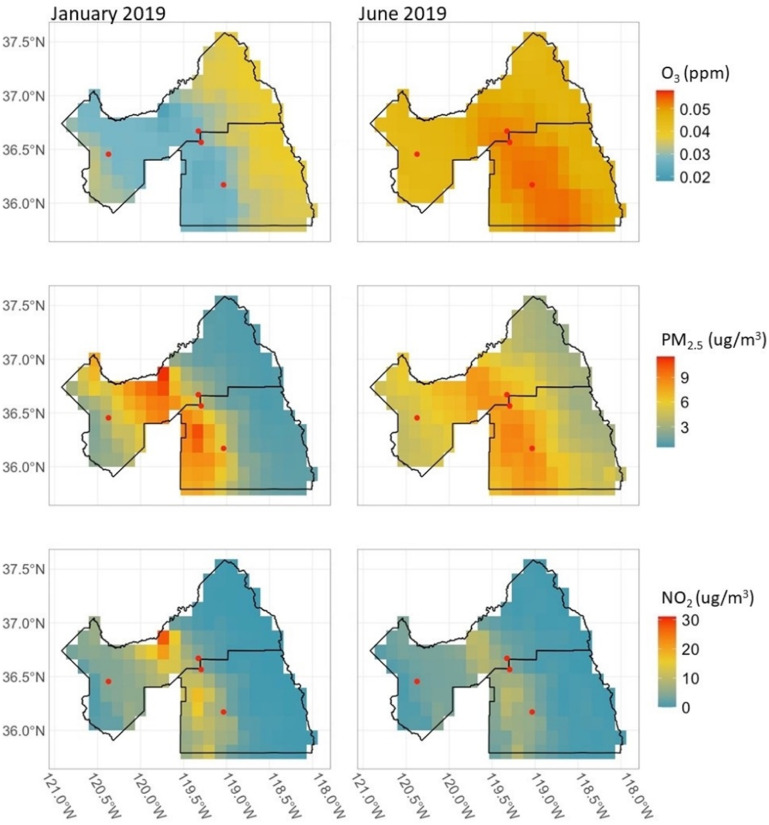
Spatial variation in the concentrations of O_3_, PM_2.5_, and NO_2_ in Fresno and Tulare Counties in January and June 2019, shown as the monthly averages based on NAAQS daily standards, as rasterized values estimated from the CMAQ model. Red points represent study community locations.

Figure 1 illustrates the spatial variation of key air pollutant concentrations—O_3_, PM_2.5_, and NO_2_ over the Fresno area in January and June 2019. Overall, PM_2.5_ and O_3_ had higher estimated concentrations in June compared to January, but NO_2_ had higher concentration estimates in January than in June (figure 1, supplementary table 2). Spatially, concentrations of PM_2.5_ were higher overall in the Central Valley region in January than in June, especially PM_2.5_ levels in the urban center of Fresno. In June, concentrations of PM_2.5_ were higher in the locations of our study communities and appeared to be more diffuse across the region. California did experience several large wildfires events in 2019 but all major fires occurred after sampling in the summer or during the fall [45].

We present the pesticide metabolite concentrations in table 2. Creatinine-normalized urinary DAP concentrations were highly skewed, with the majority of the samples having non-detectable concentrations. The mean total DAPs concentration was higher for urine samples taken in January (mean of 10.5 ng mg^−1^ of creatinine) compared to June (mean of 9.2 ng mg^−1^ of creatinine). DM metabolite concentrations followed the same seasonal pattern (mean of 10.2 ng mg^−1^ of creatinine in January and mean of 8.2 ng mg^−1^ of creatinine in June). DE concentrations were detected at higher levels from urine samples collected in June (mean of 1.0 ng mg^−1^ of creatinine in June vs. a mean of 0.3 ng mg^−1^ of creatinine in January).

**Table 2.** Summary statistics of urinary dialkyl phosphate concentrations (ng mg^−1^ of creatinine) in urinary samples taken from study participants across all study communities for January (*n* = 75) and June (*n* = 74).

Exposure	Month	Min	Q1	Median	Q3	Max	Rate of Detection % (*n*_detected_)
Total DAPs	January	0	0	0	6.6	332.6	48.0 (36)
	June	0	0	0	6.0	106.0	40.5 (30)
DM	January	0	0	0	5.6	332.6	46.7 (35)
	June	0	0	0	3.7	106.0	36.5 (27)
DE	January	0	0	0	0	23.1	1.3 (1)
	June	0	0	0	0	29.5	8.1 (6)

**Figure 2.**
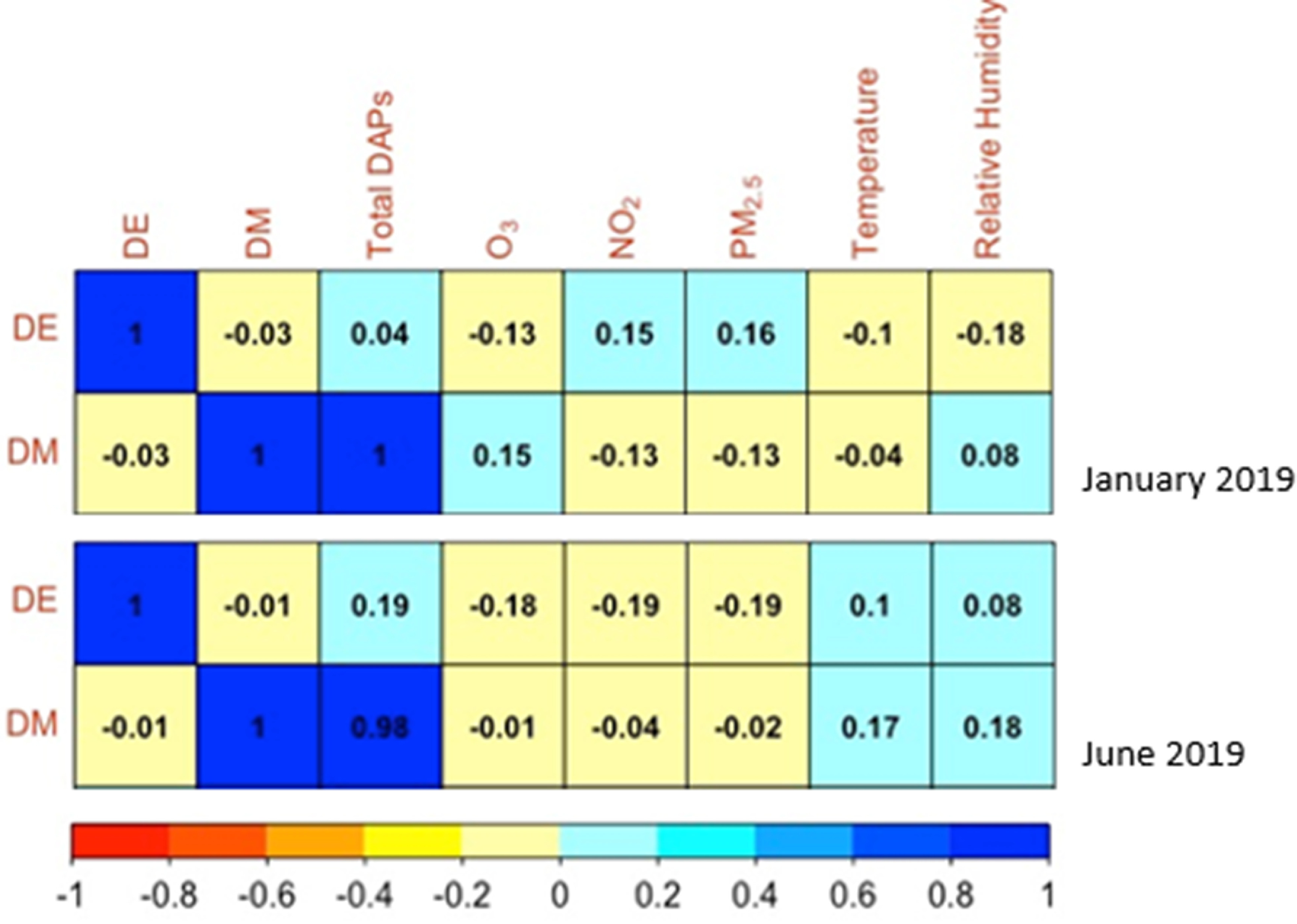
Spearman correlations among DE and DM with total DAPs, air pollutants, temperature, and relative humidity.

A Spearman correlation plot displaying the correlations between DE and DM and total DAPs, air pollutants, temperature, and relative humidity for both June and January is shown in figure 2. DE and DM were only weakly correlated with the air pollutants, temperature, and relative humidity. In June, there were negative correlations between DE and DM and the air pollutants, and positive correlations between DE and DM and temperature and relative humidity.

Creatinine-adjusted urinary LTE4 concentrations by month are presented in supplementary table 3. The results of the Wilcoxon signed-rank test indicated no statistically significant differences in urinary LTE4 concentrations between January and June (*p* = 0.51). Results of all Wilcoxon signed-rank tests for environmental exposures and LTE4 are displayed in supplementary table 4.

### 3.3. Multiple linear regression analysis

Single ECP models to estimate the relationship between the exposure and log-transformed urinary LTE4 concentrations by season are provided in table 3. Overall, the exposure-outcome associations were negative in most air pollution models and positive in pesticide models. However, confidence intervals were relatively imprecise and tended to contain the null values. Of note, an IQR increase in total DAP concentration was associated with a 4% (95% CI: 0.01, 0.07) increase in log-urinary LTE4 levels across January models. The June models also demonstrated a positive relationship between total DAPs and LTE4 levels, though confidence intervals included the null values. An IQR increase in total DM was associated with a 3% (95% CI: 0.01, 0.06) increase in log-urinary LTE4 levels for models 1 and 2, and 3% (95% CI: 0.004, 0.06) for model 3.

**Table 3.** *β* coefficients and 95% confidence intervals for multiple linear regression analyses of log-transformed urinary LTE4 and individual air pollutants and DAPs as predictor variables in crude and adjusted models.

		Model 1^a^			Model 2^b^			Model 3^c^		
Month	Exposure	*β*	95% LCI*	95% UCI**	*β*	95% LCI	95% UCI	*β*	95% LCI	95% UCI
January^e^	IQR NO_2_	−0.23	−0.49	0.17	−0.20	−0.50	0.30	−0.31	−1.10	1.07
June^f^	IQR NO_2_	−0.24	−0.45	0.04	−0.22	−0.46	0.12	0.31	−1.82	5.42
January	IQR O_3_	0.38	−0.09	1.08	0.35	−0.16	1.16	0.70	−1.12	5.86
June	IQR O_3_	−0.23	−0.44	0.06	−0.22	−0.46	0.11	0.93	−5.30	17.34
January	IQR PM_2.5_	−0.20	−0.48	0.22	−0.17	−0.49	0.35	−0.45	−1.47	1.80
June	IQR PM_2.5_	−0.24	−0.45	0.05	−0.22	−0.46	0.12	0.57	−2.54	15.07
January	**IQR Total DAPs**	**0.04**	**0.01**	**0.07**	**0.04**	**0.01**	**0.07**	**0.04**	**0.01**	**0.07**
June	IQR Total DAPs	0.02	−0.02	0.07	0.02	−0.03	0.07	0.01	−0.04	0.06
January	Total DE^d^	0.00	−0.07	0.07	0.01	−0.06	0.08	0.01	−0.06	0.09
June	Total DE^d^	−0.01	−0.04	0.03	−0.01	−0.05	0.03	−0.02	−0.06	0.02
January	**IQR Total DM**	**0.03**	**0.01**	**0.06**	**0.03**	**0.01**	**0.06**	**0.03**	**0.004**	**0.06**
June	IQR Total DM	0.02	−0.01	0.04	0.01	−0.02	0.04	0.01	−0.02	0.04

^a^ Model 1 includes individual exposure and response variables.

^b^ Model 2 includes the individual exposures and response and demographic covariates (age, sex, asthma diagnosis, current employment in agriculture, lives with someone currently employed in agriculture).

^c^ Model 3 is exposure and response plus demographic covariates, and meteorological covariates (temperature and relative humidity).

^d^ All model estimates are scaled by the IQR of the relevant exposure, with the exception of total DE, which had an IQR of 0.

^e^ January *n* = 74.

^f^ June *n* = 75.

* 95% Confidence Interval Lower Bound.

** 95% Confidence Interval Upper Bound.

### 3.4. BKMR analysis

We fit BKMR models and extracted the group and conditional PIPs for each exposure. Two groups were used for hierarchical variable selection: air pollutants (O_3_, PM_2.5_, and NO_2_) and pesticides (total DE and total DM) (table 4).

**Table 4.** Group and conditional posterior inclusion probabilities (PIPs) from BKMR, with air pollutants and pesticides as separate groups or exposure domains for hierarchical variable selection.

		January		June	
Exposure	Group	Group PIP	Conditional PIP	Group PIP	Conditional PIP
O_3_	Air pollutants	0.61	0.35	0.73	0.37
PM_2.5_	Air pollutants	0.61	0.35	0.73	0.31
NO_2_	Air pollutants	0.61	0.30	0.73	0.33
Total DE	Pesticides	0.80	0.18	0.54	0.54
Total DM	Pesticides	0.80	0.82	0.54	0.46

We observed differences in the group PIPs by season, with the pesticides group driving the association of the exposure mixture and LTE4 more during January (group PIP: 0.80), and the air pollutants group driving the association more during June (group PIP: 0.73).

Among the exposures in the air pollutant group, O_3_ and PM_2.5_ were the most important components during January (conditional PIP: 0.35 for both), and O_3_ was the most important component during June (conditional PIP: 0.37). In the pesticides group, total DM was the most important component in January (conditional PIP: 0.82), but in June, total DE contributed more to the outcome (conditional PIP: 0.54). In studies that use BKMR analysis, 0.5 is often used as a threshold for variable importance, with PIP values greater than 0.5 indicating importance of inclusion of a group or component, as well as an association with the outcome[40].

**Figure 3.**
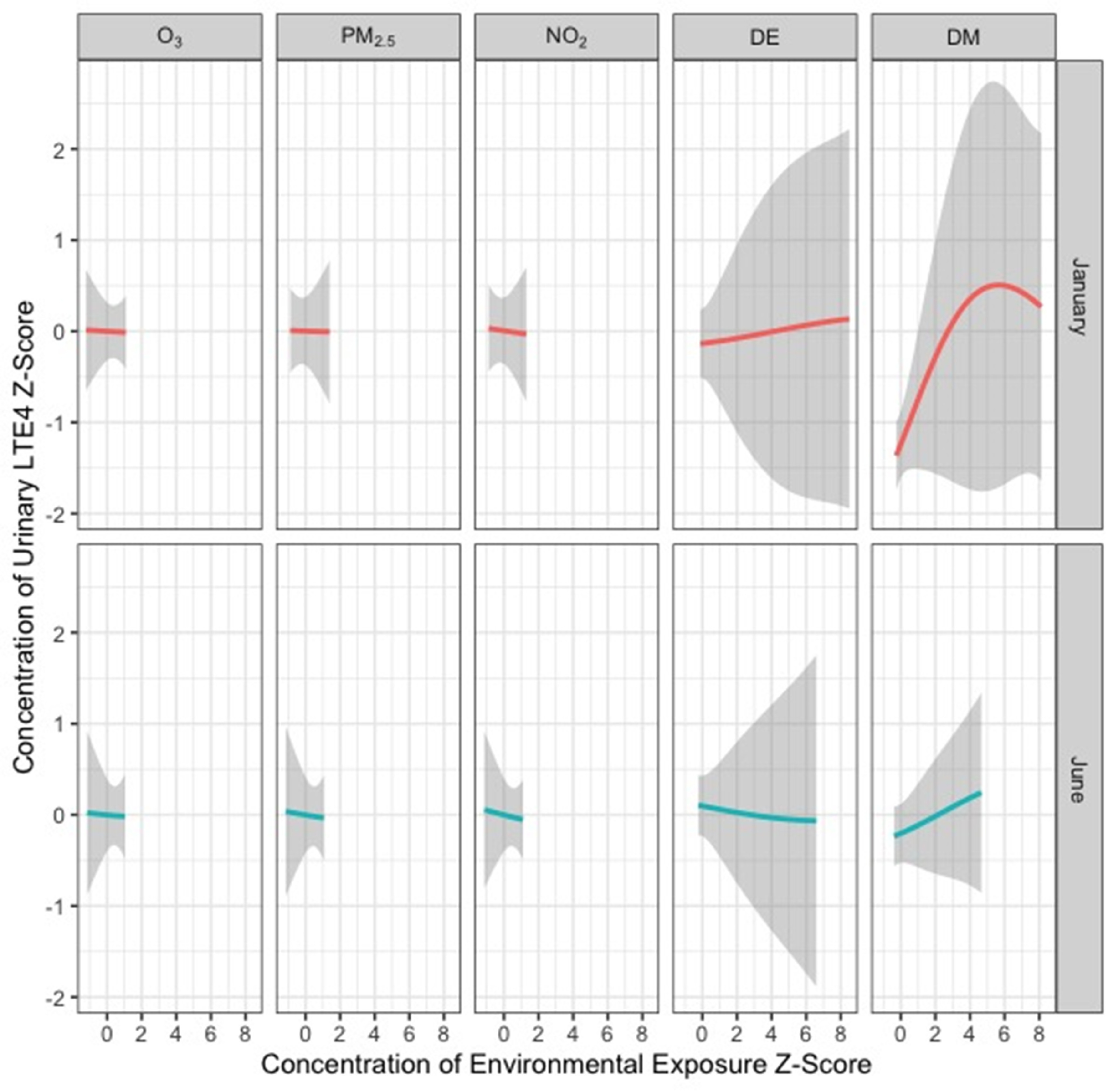
Univariate relationship between each environmental exposure and log-transformed LTE4, while holding all other exposures at their median value both in January and in June. These results were assessed using the BKMR model adjusted for age, sex, asthma diagnosis, current employment in agriculture, living with someone currently employed in agriculture, temperature, and relative humidity.

**Figure 4.**
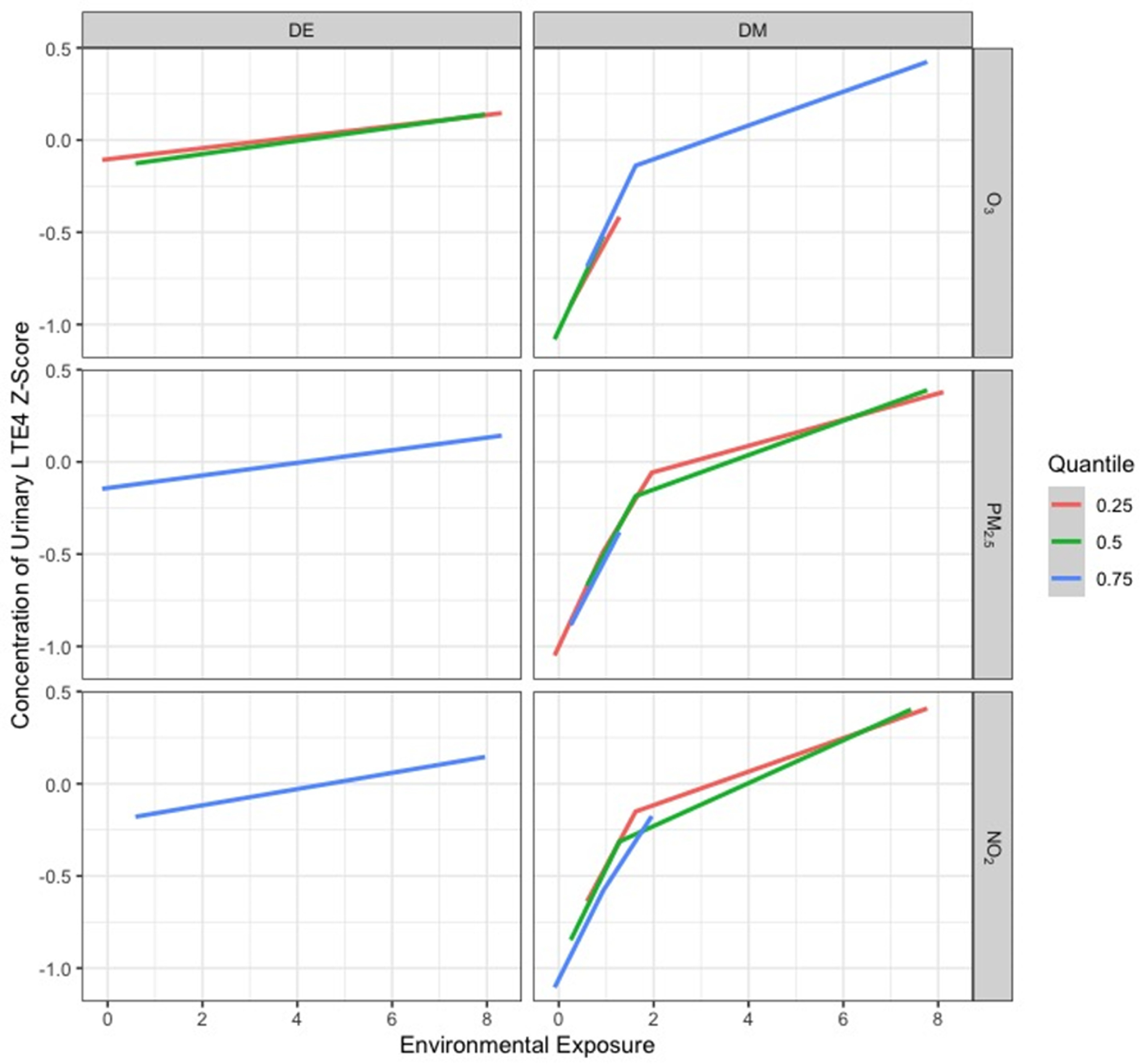
Relationship between DAPs and urinary LTE4 z-scores at the 25th, 50th, and 75th percentiles of an air pollutant exposure in January.

**Figure 5.**
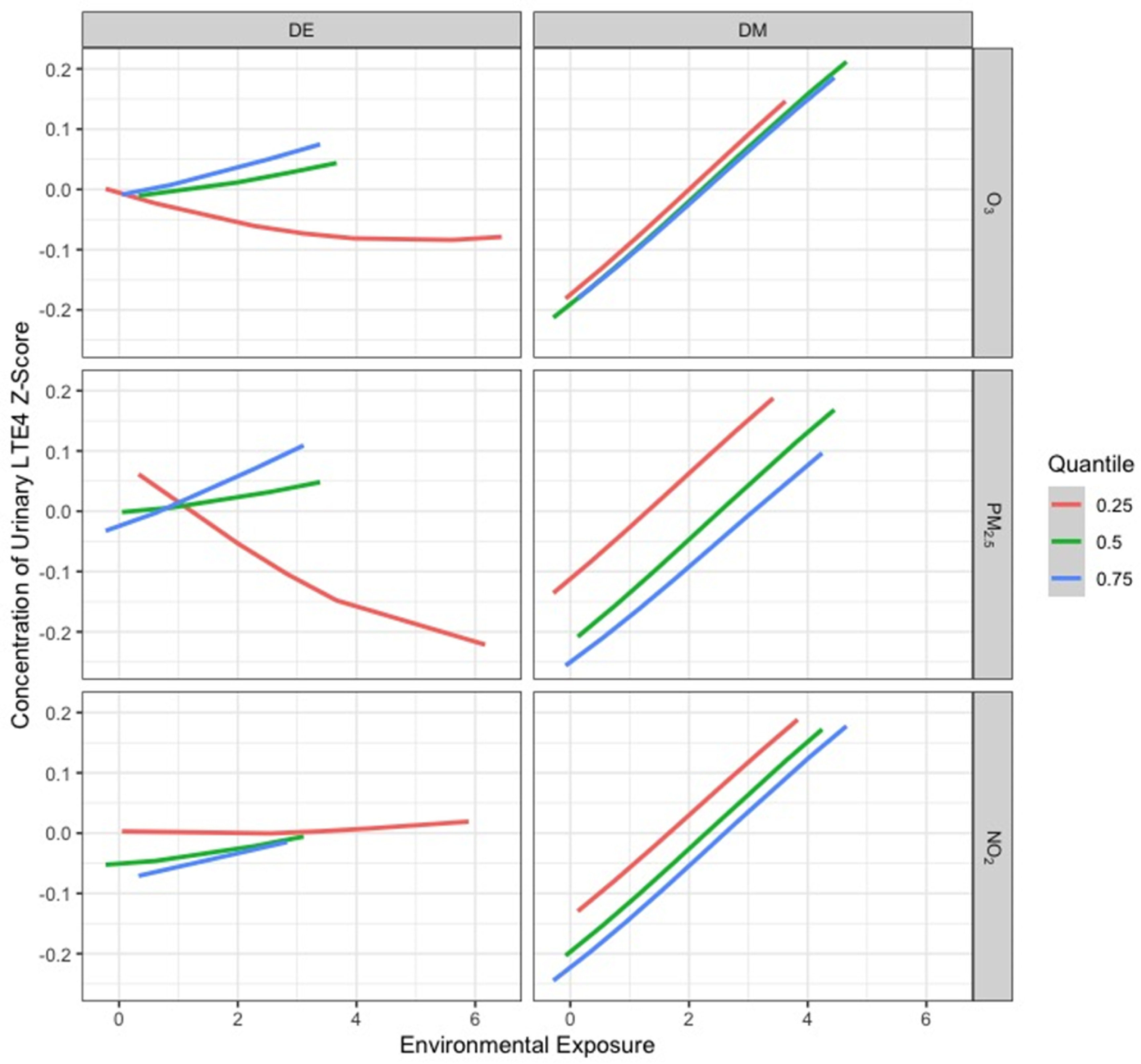
Relationship between DAPs and urinary LTE4 z-scores at the 25th, 50th, and 75th percentiles of an air pollutant exposure in June.

Figures 3–5 display cross-sectional plots of the BKMR predictor-response functions [42]. Each cross section explores the relationship between one or two exposure variables with the outcome, while setting the other exposure variables to a particular percentile [42].

Figure 3 displays the univariable relationships between each exposure variable and log-transformed urinary LTE4 concentration while holding the other exposure variables at their median values in January and June, respectively. The shaded areas represent 95% confidence bands. Total DM concentrations in January had a non-linear relationship with urinary LTE4 concentrations, with LTE4 increasing with total DM, until total DM reaches a z-score of approximately 5, after which the function decreases slightly.

For most of the other models across both time periods, we observed approximately null associations, except for positive and approximately linear associations with LTE4 z-scores for DE in January and DM in June (figure 3). A negative and approximately linear association between LTE4 z-scores for DE in June was also observed (figure 3).

Effect estimates for total DE and DM had much wider confidence intervals than the air pollutants in both January and June. This was likely due to the greater variation in DE and DM concentrations, which were taken from individuals. In contrast, only eight values of each air pollutant were used (one for each community for each season).

Figures 4 and 5 show the exposure-response relationship between log-transformed LTE4 and a given exposure at the 25th, 50th, and 75th quantiles of a second exposure, while fixing the remaining exposures at their 50th percentile, for January and June, respectively. When interpreting these types of figures, parallel lines indicate that there is no interaction between the two exposures that are being manipulated, while lines with different slopes or lines that intersect suggest that an interaction is present. For our January and June BKMR models, interaction plots show mostly parallel patterns (no interaction). The June plot (figure 5) suggests a potential interaction between total DE and O_3_, NO_2_, and PM_2.5_; the negative linear effects of total DE on LTE4 appeared to be steeper at lower O_3_, NO_2_, and PM_2.5_ levels. Complete plots showing all relationships of all environmental exposures can be found in supplementary figures 3 and 4.

## 4. Discussion

In this study, our aim was to evaluate the relationship between ambient air pollution and pesticide mixtures and respiratory inflammation. We also sought to understand how the exposure-response functions change across seasons when ECP mixtures have different compositions and properties. Mapping the air pollutant concentrations over Fresno and Tulare Counties offers evidence that the pollutant mixtures vary over space and time; likewise, pesticide application has spatial and seasonal trends in the Central Valley [3, 46].

A major finding in this study was an association between OP pesticides exposure and respiratory health, as measured by urinary levels of LTE4. Pesticide exposure has already been linked to negative health outcomes such as cancer and Parkinson’s disease [47–50], but a growing body of literature suggests that community-level pesticide exposure also affects respiratory health. Decreases in lung function (assessed using spirometry) have also been associated with exposure to pesticides. For example, results from the Canadian Health Measures Survey showed an association between increased urinary concentrations of total DAPs and reductions in lung function measures (FVC and FEV_1_) [14]. Research on the CHAMACOS cohort in Salinas, California found that increased *in utero* exposure to OPs was associated with decreases in FEV_1_ and FVC for children at age 7 [13, 51].

Currently, the majority of the literature on pesticides and respiratory health is based on occupational studies. Agricultural workers are at an especially high risk for asthma and other respiratory diseases due to their frequent exposure to bioaerosols, dust, and pesticides [11]. Research using data from the Agricultural Cohort Study in Iowa and North Carolina has suggested a dose-dependent association between OP pesticides and wheeze symptoms[11]. Other negative respiratory health outcomes including cough, chest tightness, throat irritation, dyspnea, and difficulty breathing have also been observed in workers exposed to pesticides in multiple farm settings in Croatia, Iowa, and North Carolina [7, 52, 53].

Our study demonstrates an association between pesticide exposures and biomarkers of respiratory inflammation among members of Central California agricultural communities. This association was significant in the month of January but not significant in the month of June. More studies need to be conducted to further evaluate such an association, but our study adds to this growing body of research.

Another implication of our study is that LTE4 may be an effective biomarker for respiratory inflammation in a general population that has low prevalence of asthma. In our study there was a statistically significant association between the levels of total DAPs and urinary LTE4, and total DM and urinary LTE4 in January, adjusting for asthma status. Currently, using LTE4 as a biomarker is an accepted non-invasive method of assessing respiratory inflammation among people who have asthma as it is an indirect indicator of a lipid mediator known to play a central pathophysiological role in asthma (i.e. lung cys-LT) [31]. Biomarkers have proven effective in epidemiologic studies assessing the relationship between air pollutants, pesticides, and respiratory outcomes. Urinary leukotriene E4 (LTE4) is a validated, sensitive, and non-invasive biomarker of respiratory health among people with asthma [54], and has been used as a biomarker of asthma-related respiratory symptoms in studies where both air pollutants and pesticides are the exposures of interest [31, 55]. However, more research is needed to clarify the effectiveness of urinary LTE4 as a biomarker for respiratory disease in people without asthma. A study by Makena *et al* suggested that levels of urinary LTE4 declined in smokers who switched from combustible to noncombustible tobacco products [56]. An additional study documented increased urinary LTE4 levels in workers exposed to increased volatile organic compounds (VOCs) [57]. Further, urinary LTE4 measurements were found to be associated with occupational VOC exposure in workers without asthma [57]. In a metabolomics analysis of pregnant women, annotations in the leukotriene metabolism pathway demonstrated increased abundance in the high air pollution exposure group compared to a low air pollution exposure group, suggesting that LTE4 may be a marker of inflammation associated with adverse environmental exposures in groups without asthma [58].

These studies suggest that there may be a likely pathway linking adverse inhalational exposures and LTE4 increases in populations without asthma. Implementation of LTE4 as a biomarker for respiratory inflammation in populations without asthma requires additional research, as it has important limitations. Mainly, elevated levels of urinary LTE4 can occur with exacerbation of other atopic diseases [59], including some conditions that are also associated with air pollution and OP pesticides such as dermatitis [60, 61].

Finally, we observed non-linear associations between exposures in a mixture and a respiratory health outcome, albeit not statistically significant. Currently, the volume of literature on environmental mixtures is sparse. Researchers in Colombia observing farmers’ exposure to pesticide mixtures demonstrated a significant association between exposure and asthma [62]. In addition, that study demonstrated associations between different chemical mixtures and various respiratory disorders, such as the flu, thoracic pain, allergic rhinitis, and obstructed patterns in spirometry [62]. In a study similar to ours conducted in Washington State, Benka-Coker *et al* observed that increasing total mixture levels of PM_2.5_, O_3_, and OPs was associated with increased levels of urinary LTE4 [40]. Our study showed that the exposure-response relationship between three air pollutants and DAPs as a mixture is different depending on the season and the different levels of pollutants within the mixture.

Environmental mixtures are an important new frontier in environmental epidemiology, and BKMR is a viable tool to study and assess risk of exposure to mixtures. A particular strength of using BKMR is that the method allows for non-linearity and more flexibility in the relationships between exposures and health outcomes compared to traditional regression models. The BKMR mixture analysis in our study revealed non-linear associations that linear regression models would not capture. For future research this could indicate that linear models may not always be appropriate for studying joint environmental exposures.

Despite the advantages of BKMR and mixtures research, Sexton *et al* addresses some limitations of studying cumulative risk to environmental mixtures: relatively little is known about the duration, magnitude, frequency, timing, and exposure to environmental mixtures; there is little evidence about whether the effects of mixture components are antagonistic, synergistic, or additive; and the mechanisms of toxicity in mixture components are poorly understood [63]. Additionally, pollutants may have different etiological windows associated with certain health outcomes. For example, Raanan *et al* indicated that respiratory health effects among children are associated with both prenatal and postnatal exposure to OP pesticides, but stronger associations were observed in children with observed urinary metabolites detected between 0.5 and 5 years of age than in utero, measured by maternal urinary DAPs [51].

There are some important limitations to discuss in this study. One is that the sample size is small; 74 participants in January provided samples, and 75 provided samples in June. Another limitation of this study is that we used an ecological measurement for air pollution exposure (community level), but an individual measurement for pesticide exposure. This had the potential to introduce Berkson error for the former. Classical error is also probable in our individual pesticide samples, as DAPs represent short-term exposure. Further, urinary DAPs do not indicate the route of exposure or the specific types of OPs to which the individuals in the study were exposed. Because DAPs are excreted from the body quickly after exposure, they also do not indicate duration of exposure. Information such as the number of years participants worked in agriculture, or how long they had lived in Fresno or Tulare Counties might be a more useful estimate of exposure, in terms of duration. It is likely that participants who had been working with pesticides longer would have a higher cumulative pesticide exposure. In addition, participants who had lived in Fresno or Tulare Counties longer would have a higher cumulative exposure to air pollutants.

We observed higher levels of DAPs in January than in June, despite the fact that June is the pesticide application season in Central California. Previous work by Kuiper *et al* documented that OP pesticide residues can persist in indoor environments for long periods of time due to a lack of light, water and microbial degradation, thus extending the half-life of residues [21].

## 5. Conclusion

In a community-based study where ambient air pollution and pesticide application are high, we found that some urinary metabolites of OP pesticides (DAPs and DM) were positively associated with a urinary marker of respiratory inflammation in both January and June; DE showed a negative and non-significant association in June. Since the prevalence of asthma was low in this population, we suggest that the potential for LTE4 to be used as a biomarker for respiratory inflammation in individuals who do not have asthma should be further explored. We also suggest that interactions between different air pollution and pesticide exposures exist, some are non-linear, and these mixtures had different relationships with the health outcome in application season and non-application season. These findings demonstrate that some exposure response relationships require methods other than linear regression to capture them. To effectively study an environmental mixture of air pollutants and pesticides, future research should strive to enroll larger sample sizes and individual air pollutant measurements should also be considered.

## Data Availability

The data cannot be made publicly available upon publication because they contain sensitive personal information. The data that support the findings of this study are available upon reasonable request from the authors.
